# Enzymes of early-diverging, zoosporic fungi

**DOI:** 10.1007/s00253-019-09983-w

**Published:** 2019-07-15

**Authors:** Lene Lange, Kristian Barrett, Bo Pilgaard, Frank Gleason, Adrian Tsang

**Affiliations:** 1 Bioeconomy, Research & Advisory, Karensgade 5, Valby, DK-2500 Copenhagen, Denmark; 2 Protein Chemistry and Enzyme Technology, Department of Biotechnology and Biomedicine, Technical University of Denmark, Søltofts Plads 221, DK-2800 Kgs. Lyngby, Denmark; 3 School of Life and Environmental Sciences, University of Sydney, Sydney, 2006 Australia; 4 Centre for Structural and Functional Genomics, Concordia University, Montreal, QC, H4B1R6 Canada

**Keywords:** Early-diverging fungi, Zoosporic aerobic and anaerobic fungi, Functional genomics, Secretome enzyme composition, Secretome evolution, Biomass-degrading enzymes, Biotech potential

## Abstract

The secretome, the complement of extracellular proteins, is a reflection of the interaction of an organism with its host or substrate, thus a determining factor for the organism’s fitness and competitiveness. Hence, the secretome impacts speciation and organismal evolution. The zoosporic *Chytridiomycota*, *Blastocladiomycota*, *Neocallimastigomycota*, and *Cryptomycota* represent the earliest diverging lineages of the Fungal Kingdom. The review describes the enzyme compositions of these zoosporic fungi, underscoring the enzymes involved in biomass degradation. The review connects the lifestyle and substrate affinities of the zoosporic fungi to the secretome composition by examining both classical phenotypic investigations and molecular/genomic-based studies. The carbohydrate-active enzyme profiles of 19 genome-sequenced species are summarized. Emphasis is given to recent advances in understanding the functional role of rumen fungi, the basis for the devastating chytridiomycosis, and the structure of fungal cellulosome. The approach taken by the review enables comparison of the secretome enzyme composition of anaerobic versus aerobic early-diverging fungi and comparison of enzyme portfolio of specialized parasites, pathogens, and saprotrophs. Early-diverging fungi digest most major types of biopolymers: cellulose, hemicellulose, pectin, chitin, and keratin. It is thus to be expected that early-diverging fungi in its entirety represents a rich and diverse pool of secreted, metabolic enzymes. The review presents the methods used for enzyme discovery, the diversity of enzymes found, the status and outlook for recombinant production, and the potential for applications. Comparative studies on the composition of secretome enzymes of early-diverging fungi would contribute to unraveling the basal lineages of fungi.

## Introduction

Functional studies of the early-diverging lineages of fungi have been driven by the urge to understand the basis for the devastating chytridiomycosis in amphibians and the role of fungi in the rumen microbiome especially in the digestion of fibrous carbohydrates. The collaborative efforts of the scientific community to advance the 1000 fungal genome project have also been a significant enabler for genomic and ancillary studies of a broader diversity of zoosporic fungi. Unraveling the basal lineages of fungal phylogeny is a prerequisite to understanding the evolution of fungi. Early-diverging fungi have acquired diverse lifestyles. It is therefore important for studies of these organisms to take into account their genotypic and phenotypic characteristics, as well as the environment in which they dwell. Another key aspect of investigation is the composition and evolution of the secretome, referred here as the set of proteins secreted to the extracellular space. The secretome is a reflection of the interaction of an organism with its host or substrate and with the environment, and thus, in an integrated manner, the secretome impacts speciation.

Early-diverging fungi thrive in many different habitats: anaerobic and aerobic, terrestrial and aquatic. They have specialized into many different lifestyles: biotrophic and saprotrophic; symbiotic, parasitic, and pathogenic; and they metabolize organic matters of animal, plant, algal, and fungal origin. More specifically, the early-diverging fungi have been shown to digest most major types of biopolymers: cellulose, hemicellulose, pectin, chitin, and keratin. The (aquatic) zoosporic fungi are poor in enzymes acting on the polymer lignin. (Unlike that found in the terrestrial biosphere, lignin is a plant cell wall component of minor abundance in aquatic habitats). Based on their broad biomass-degrading capabilities, it is to be expected that the early-diverging fungi in general possess a wide range of secreted, metabolic enzymes. However, there are also members with reduced repertoires of extracellular carbohydrate-active enzymes (CAZymes), reflecting specialized forms such as pathogens of animals, which lack cell walls. Yet, the early-diverging fungi are an understudied and industrially under-exploited reservoir of novel and possibly unique enzymes and lifeforms. The objective of this review is adopting a holistic approach to discussing the enzymes of early-diverging, zoosporic fungi (across all four phyla), to inspire further studies of these intriguing and diverse organisms. For this review, we adopt the classification of the 2014 MycoCosm (Grigoriev et al. 2014). The phyla to be discussed are *Blastocladiomycota*, *Chytridiomycota*, *Neocallimastigomycota*, and *Cryptomycota*, all zoosporic and all in one way or another connected to water. *Microsporidia* and *Kickxellomycotina* also belong to the early-diverging lineages of fungi. However, they are not zoosporic and with only limited information available about their adaptation to parasitic life on hosts with no cell walls. Thus, the *Microsporidia* and *Kickxellomycotina* are considered beyond the scope for this review, only used to add evolutionary perspectives to the overview here on the enzymes of the zoosporic fungi. Recently, using whole proteomes to determine evolutionary phylogeny of fungi, Choi and Kim (2017) placed *Neocallimastigomycota* outside the Fungal Kingdom and as a phylum of protozoans. Furthermore, a new MycoCosm recently released by the Joint Genome Institute (JGI) places *Neocallimastigomycetes* under the phylum *Chytridiomycota* (https://genome.jgi.doe.gov/mycocosm/home). As demonstrated by these recent analyses, the classification of early-diverging, zoosporic fungi remains an active area of investigation. Additional studies on the genomes, proteomes, and secretomes of these organisms should provide further insights into the evolution of early-diverging fungi.

## Morphology, habitats, and isolation

Classical studies of zoosporic fungi included meticulous observations of substrate affinities and interactions, as well as attention to the morphology of fungal structures. There are many structures of significance for enzymatic interactions, which modify their hosts and substrates. Among the zoosporic fungi, it is the rhizoids of both the monocentric and the polycentric, anaerobic, and aerobic chytrids that are thought to be the structures from which enzymes are primarily secreted (Sparrow 1960; Karling 1977). Since secretion mechanism for enzymes among the early-diverging, zoosporic fungi is incompletely studied, no evidence has been provided for a secretion mechanism different from what is known of Dikarya fungi (Read 2011).

In contrast to the aerobic chytrids, the analysis of polysaccharide-degrading enzymes has been an integral part of early studies on anaerobic fungi isolated from herbivores (reviewed in Borneman and Akin 1994, and recently by Gruninger et al. 2014). Orpin recognized the rumen microorganisms previously called flagellated protozoae as fungi (Orpin 1977). Notably Orpin l. c. also noticed the marked attack on plant fibers and recognized it as fungal degradation. Heath et al. (1983) validated the species characteristics of the genus *Neocallimasti*x and assigned it to a new lineage, including a new family, order, and class, the *Neocallimastigomycetes*.

**Fig. 1:**
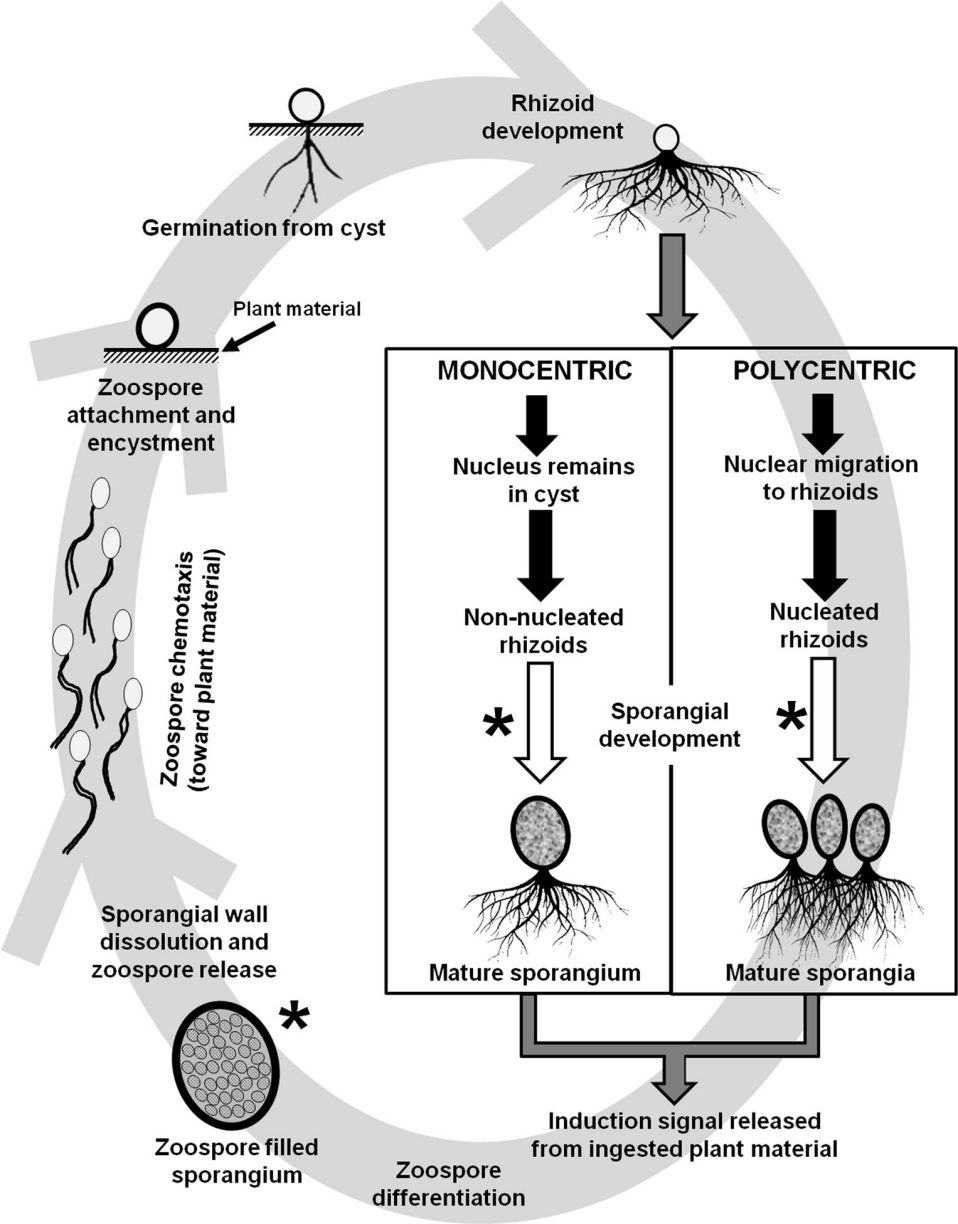
Summary of the anaerobic fungal life cycle. The stages in the life cycle where “resistant” structures (that have been reported to date) may be formed are also indicated (marked by asterisk). With courtesy of FEMS Microbiology Ecology, Volume 90, Issue 1, October 2014, Pages 1–17, 10.1111/1574-6941.12383

Morphological studies of how the anaerobic zoosporic fungi invade lignocellulosic substrate in the rumen have been reviewed in Gruninger et al. (2014). The rhizomycelium anchors the anaerobic rumen fungi to their substrates (see Fig. 1, schematic life cycle from Gruninger et al. 2014). At the same time, the rhizoids and rhizomycelium physically penetrate and enzymatically degrade the plant material. This process provides sufficient energy and nutrients for the rumen fungi to invade the undamaged plant cells and thus makes more of the food available for further microbial digestion.

Technologies for isolation of early-diverging fungi have exploited the aquatic nature and substrate affinities of these fungi, and thereby, baiting methods were developed (Sparrow 1960; Karling 1977). Minute amounts of sediment or soil samples were submerged in water, and a variety of baits were added: pollen grains to ensnare plant cell wall–degrading organisms; cellophane for cellulose degraders; and snakeskin and/or defatted hair for keratin degraders. Interestingly, blond baby hair showed best performance to attract keratin-degrading zoosporic fungi (personal communication, Sparrow 1960). Such baiting materials allowed light microscopic studies by simply mounting the bait directly to visualize the sporangia, the rhizoidal structures, and the process of discharge of uniflagellate zoospores. Emerson and his students developed a suite of methods for laboratory isolation and cultivation of zoosporic fungi (Fuller 1978). Of importance to successful aquatic cultivation of zoosporic fungi, Fuller and co-workers developed a laboratory recipe for making optimized “pond water” (Fuller, l.c.). Still, the inherent difficulties in culturing most species of zoosporic fungi in the laboratory have been a major reason for the dearth of available information about their enzyme activities compared, for example, with the zoosporic Oomycetes where several enzyme activity studies are available from, e.g., *Phytophthora* spp. (Ospina-Giraldo et al. 2010).

## Overview of genome-sequenced zoosporic fungi

To date, the genomes of 14 species of aerobic and five anaerobic early-diverging fungi have been sequenced; representing all four phyla, the *Chytridiomycota*, *Blastocladiomycota*, *Neocallimastigomycota*, and *Cryptomycota* (Table 1). Besides species name and taxonomic classification, Table 1 includes descriptions of substrate affinity and lifestyle, and the digestive enzyme profiles of the genome-sequenced species. The CAZyme profile of each species is based on JGI annotation (MycoCosm) except for the species marked with an asterisk, which have been annotated by the new peptide-based annotation method CUPP (conserved unique peptide pattern; Barrett and Lange 2019). The CAZyme profile is divided into auxiliary activity (AA) enzymes, carbohydrate esterases (CE), glycoside hydrolases (GH), glycosyltransferases (GT), and polysaccharide lyases (PL). A summary number of proteases based on JGI annotation is given in the right-hand column of Table 1. The most striking message taken from the enzyme profile presented in Table 1 is the differences in the enzyme complement among the zoosporic fungi. Compared with the pathogenic or parasitic fungi, the biomass decomposers are rich in CAZymes involved in polysaccharide-degrading enzymes. The saprotrophic *Homolaphlyctis polyrhiza* is the exception in that it possesses relatively few CAZymes. *Batrachochytrium dendrobatidis* and *H. polyrhiza* are closely related species (Joneson et al. 2011). Though they have adopted different lifestyles, one a parasite and the other saprotroph, they possess similar number of CAZymes.

**Table 1:** Summary of substrate, lifestyle, and enzyme profile of early-diverging fungi with sequenced genome. Listed are 14 aerobic species from three different phyla and five species of anaerobic rumen fungi from the phylum *Neocallimastigomycota*. The collection of CAZymes and proteases of these 19 species are shown in right-hand columns. Enzyme annotations to major groupings of CAZymes are based on JGI (MycoCosm) annotations: *AA* auxiliary activity enzymes, *CE* carbohydrate esterases, *GH* glycoside hydrolases, *GT* glycosyltransferases, and *PL* polysaccharide lyases. Similarly, the number of protease genes was obtained from JGI MycoCosm genome resource, the annotations of which were based on the Merops database. For four of the genomes, where no JGI annotation was available, the CAZy-annotations were done by CUPP (conserved unique peptide patterns) in the strict, conservative mode (indicated by an asterisk). Using CUPP with more relaxed parameters opens for finding more (and more diverse) hits, however, may require some manual inspection. The genome of *S. endobioticum* is not analyzed due to unexpectedly small genome size. Further studies of this genome are needed prior to functional annotation

Taxonomy		Substrate and life style	CAZy families						Merops proteases	Accession	CUPP
Species	Order		AA	CE	GH	GT	PL	Sum			
*Blastocladiomycota*											
*Allomyces macrogynus*	*Blastocladiales*	In tropical soils and slow-moving rivers and ponds	15	15	96	162	2	290	432	GCA_000151295.1	490
*Catenaria anguillulae*	*Blastocladiales*	Facultative parasite on nematodes; in soil	11	15	35	84	1	146	-	GCA_002102555.1	243
*Chytridiomycota*											
*Caulochytrium protostelioides*	*Caulochytriales*	Obligate parasite on deuteromycetous fungi	11	4	41	52	0	108	152	GCA_003615045.1	659
*Rhizoclosmatium globosum*	*Chytridiales*	Saprotrophic, on insect chitinaceous exuviates	13	21	202	197	9	442	360	GCA_002104985.1	574
*Gonapodya prolifera*	*Monoblepharidales*	Aquatic, saprotrophic, on decaying fruits	16	29	139	96	9	289	341	GCA_001574975.1	300
*Blyttiomyces helicus*	–	Growing on algae and pollen; parasitic on chytrids	23	24	90	73	0	210	256	GCA_003614705.1	174
*Rhizophlyctis rosea*	*Rhizophlyctidales*	Biomass degrader, in agricultural soils	45	42	144	73	12	316*	-	GCA_002214945.1	370
*Batrachochytrium dendrobatidis*	*Rhizophydiales*	Pathogen on vertebrates (amphibian; frogs)	8	23	45	83	4	163	314	GCF_000203795.1	140
*Batrachochytrium salamandrivorans*	*Rhizophydiales*	Pathogen on vertebrates (amphibian; salamanders	3	15	32	54	2	106*	-	GCA_002006685.1	132
*Homolaphlyctis polyrhiza*	*Rhizophydiales*	Saprotrophic, on decaying plant debris	9	5	48	77	2	141	184	GCA_000235945.1	165
*Spizellomyces punctatus*	*Spizellomycetales*	Ubiquitous, in soils, decomposing pollen	12	18	63	84	2	179	210	GCF_000182565.1	173
*Synchytrium endobioticum*	*Synchytriales*	Pathogen, on potatoes; cause potato warts disease	3	0	3	3	0	9*	-	GCA_001399855.1	9
*Cryptomycota*											
*Paramicrosporidium saccamoebae*	–	Freshwater, soils and marine sediments	0	0	14	37	0	51*	-	GCA_002794465.1	60
*Rozella allomycis*	–	Endoparasite on fungi (*Allomyces* spp.)	6	7	24	52	0	89	143	GCA_000442015.1	52
*Neocallimastigomycota*											
*Anaeromyces robustus*	*Neocallimastigales*	Rumen fungi; degrading lignocellulose	0	121	261	127	11	520	331	GCA_002104895.1	381
*Neocallimastix californiae*	*Neocallimastigales*	Rumen fungi; degrading lignocellulose	0	213	548	190	82	1033	443	GCA_002104975.1	997
*Pecoramyces ruminatium*	*Neocallimastigales*	Rumen fungi; degrading lignocellulose	0	119	379	109	30	637	495	GCA_000412615.11	032
*Piromyces finnis*	*Neocallimastigales*	Rumen fungi; degrading lignocellulose	0	92	279	106	15	492	228	GCA_002104945.1	384
*Piromyces sp. E2*	*Neocallimastigales*	Rumen fungi; degrading lignocellulose	0	139	472	104	32	747	395	GCA_002157105.1	584

## Enzyme discovery in early-diverging fungi

As early as 1994, Borneman and Akin reviewed a broad list of enzyme activities documented or assigned to the rumen fungi. Dijkerman et al. (1996) described in detail the diversity of rumen enzymes, especially those involved in lignocellulose degradation. Harhangi et al. (2003) pioneered DNA-based enzyme discovery in rumen fungi. Hodrova et al. (1997) documented that the cellulolytic endoglucanase activity of both monocentric and polycentric rumen fungi was secreted. For beta-glucosidase activity, most of the activity for the polycentric species, however, were found to be bound to the cell surface. Hodrova (l.c.) reported higher levels of endoglucanase activity in the polycentric compared to the monocentric species. They concluded that the polycentric species are more efficient than the monocentric species in the utilization of cellulosic substrates (either microcrystalline cellulose or alfalfa hay). However, the monocentric species has higher beta-glucosidase activity. These findings prompted the postulation that the different rumen fungi may have different and complementary roles, rather than being solely competitors, in the utilization of lignocellulosic substrate (Gruninger et al. 2014). Industry has also participated in the studies of enzymes from anaerobic fungi (Genencor International) and the aerobic chytrid, *Rhizophlyctis rosea* (Novozymes, GH45 patent; Kauppinen et al. 1998). In the last 10 years, genome sequencing has brought the studies of secretomes of the early-diverging fungi into a new era. Such studies have since revealed the enormous enzyme diversity, and possibly uniqueness, found in the secretomes of zoosporic fungi.

Enzyme discovery studies of early-diverging fungi in the post-genomics era have followed different approaches, depending on the objectives of the studies. Chang et al. (2015) showed how fungi are involved in shaping the earliest terrestrial ecosystems by analyzing the phylogenomics of pectin-active enzymes of early-diverging fungi (discussed later). Taking a taxonomic approach, Letcher et al. (2006) combined the use of morphology, zoospore ultrastructure, and SSU/LSU rDNA sequence analysis in defining a new order, the *Rhizophydiales.* In performing this task, they also provided valuable observations, as seen from an enzyme secretome point of view, regarding rhizoid structures. The rhizoids are the fungal morphological structure for secretion of substrate-metabolizing enzymes and for anchoring the thallus to the substrate (or host). Notably, the Letcher et al. (2006) paper includes informative micrographs of rhizoidal structures of early-diverging fungi. The *Rhizophydiales*, in which three of the genome-sequenced species (*B. dendrobatidis*, and *B. salamandrivorans* and *Homolaphlyctis polyrhiza*) belong (Table 1), have been described as having a variety of lifestyles: soil-inhabiting, saprophytic on plant materials (pollen); biotrophic on the hyphal *Oedogoniomyces*; or pathogenic on frogs and salamanders. Resolving the taxonomy of the *Rhizophydiales*, Letcher et al. (2006) provided a good basis for further studies of the severe chytridiomycosis epidemic of amphibians.

The discovery of a chytrid fungus as the causative agent of a serious disease that threatens amphibian biodiversity and populations has led to extensive studies of its pathogenesis. These studies included defining the enzyme activities of the *B. dendrabatidis* secretome. Symonds and co-workers adopted an experimental approach to identify the spectrum of enzyme activities (Symonds et al. 2008). They used the colorimetric API ZYM test kit (bioMerieux sa, 69280, Marcy-l’Ètoile, France) to identify multiple enzyme activities in the *B. dendrobatidis* secretome. The most dominant enzyme activities identified are proteolytic and esterase activities. Keratinolytic activity was observed along with activities of esterase (C4-active), esterase lipase (C8-active), lipase (C14-active), alpha-glucosidase, and beta-glucosidase. In another research effort, Berger et al. (2016) focused on enzyme activities from *B. dendrobatidis* during interaction with its host. They confirmed proteases, as putative virulence factors, and lipases to be the two most prominent types of enzyme activities. Among their results was the identification of adhesin and CRN-like effector genes, both of which have been hypothesized to be components of pathogenesis of this species.

Another approach to *B. dendrobatidis* enzyme discovery was the study of Joneson et al. (2011). They addressed the evolutionarily and biologically important question: could genome comparison between two closely related fungi, *H. polyrhiza* and *B. dendrobatidis*, be used to elucidate the transition from a saprotrophic lifestyle to a pathogen. Joneson and co-workers identified that the set of 1974 genes specific to *B. dendrobatidis* is enriched for genes encoding proteases, lipases, and microbial effectors as compared to *H. polyrhiza*. These authors were further able to conclude that expansion of gene diversity occurred after the divergence of *B. dendrobatidis* and *H. polyrhiza.* Recently, differences in the portfolio of carbohydrate-active enzymes of *B. dendrobatidis* and *H. polyrhiza* have been elucidated by Lange et al. (2018) based on peptide-based functional annotation of the two genomes (Busk et al. 2017). *Homolaphlyctis polyrhiza* was found to possess GH35 beta-galactosidase (3.2.1.23) and GH36 alpha-galactosidase (EC 3.2.1.22) while *B. dendrobatidis* was found not to have these two enzymes. However, *B. dendrobatidis* was found to have GH3 beta-glucosidase (EC 3.2.1.21) and AA1 laccase (EC 1.10.3.2). In addition, both species have been shown to share the same three enzyme functions for degrading starch (EC 3.2.1.3, EC 3.2.1.20, and EC 3.2.1.28), the enzyme for degrading mannan (EC 3.2.1.113, represented by GH47), and the two activities of cell wall-modifying enzymes, chitinase (EC3.2.1.14) and chitin-deacetylase (EC 3.5.1.41). In addition to this are *H. polyrhiza* hasalpha-mannosidase (EC 3.2.1.24) and beta-mannosidase (EC 3.2.1.25) as well as a predicted GH20 beta-*N*-acetyl hexoaminidase (EC 3.2.1.52).

James and coworkers (James et al. 2013) found shared signatures of parasitism between the early-diverging lineages *Cryptomycota* and *Microsporidia*. Both phyla have chitin synthase and both have chitin in their cell walls. In this study, the occurrence of similar enzymes and cell wall materials were used to suggest that the *Cryptomycot*a and the *Microsporidia* share a common endoparasitic ancestor, with the clade that includes the *Cryptomycota* and the *Microsporidia* unified by a chitinous cell wall. Lange et al. (2018) found AA11 LPMO (lytic polysaccharide monooxygenase) in *R. allomycis* (Tables 2 and 3). The AA11 LPMO has been suggested to be involved in degradation of the chitin polymer (Forsberg et al. 2016). For *R. allomycis*, AA11 could be considered to be involved in penetrating the chitinous cell wall of their host (*Allomyces spp*); however, it could also be involved in modifying its own chitinous wall. Furthermore, *R. allomycis* harbors a set of genes, encoding mostly enzymes that degrade storage carbohydrates, e.g., starch-degrading enzymes (EC 3.2.1.20 and EC 3.2.1.28) (Tables 2 and 3). It is noteworthy that this set of starch-degrading enzymes and chitinase (EC 3.2.1.14) are found to be shared among the 12 genome-sequenced zoosporic fungi (see Tables 2 and 3). Tables 2 and 3 have been modified from Lange et al. (2018). Notably, in Lange et al. (2018), use of secretion prediction tools, such as SignalP or Phobious, was de-selected after test runs. None of the prediction tools has been developed for (or trained on) enzymes from early-diverging, zoosporic fungi, and test runs showed that using these secretion prediction tools resulted in high level of false negatives.

**Table 2:** Comparative analysis of enzymes from four phyla of early-diverging, zoosporic fungi. *Chytridiomycota species* The left hand column lists the enzyme functions found in the respective genomes, predicted to EC enzyme classification as well as EC functional description by peptide-based functional analysis (herein the peptide-based program, Hotpep). The table is modified from, and based on the data published in, Lange et al. (2018). The EC functions are grouped according to the type of substrate the predicted enzymes act on: plant cell wall carbohydrates (cellulose, hemicellulose, and pectin) and storage and fungal cell wall carbohydrates (starch, mannan, chitin)

		Chytridiomycota											
EC #	Function description	*Rhizophlyctis rosea*				*Spizellomyces punctatus*	*Rhizoclosmatium globosum*		*Homoloaphlyctis polyrhiza*	*Batrachochytrium dendrobatidis*	*Gonapodya prolifera*		
Cellulose													
3.2.1.4	Endo-1,4-β-D-glucanase	2 GH9	2 GH7	1 GH45	8 GH5	1 GH9					1 GH9	3 GH45	3 GH26
3.2.1.21	β-glucosidase	4 GH3	3 GH1			1 GH3	3 GH3	3 GH1		1 GH3	3 GH3		
3.2.1.91	cellulose_1,4-β-cellobiosidase (non-red_end)	6 GH6											
3.2.1.176	cellulose_1,4-β-cellobiosidase_(red_end)	3 GH7											
AA9	LPMO	24 AA9									2 AA9		
1.1.99.18	cellobiose_dehydrogenase_(acceptor)	1 AA8					1 AA3				2 AA3		
1.1.99.29	pyranose_dehydrogenase_(acceptor)	1 AA3											
Hemi-cellulose													
3.1.1.6	acetylesterase	2 CE16	2 CE1										
3.1.1.72	Acetylxylan_esterase	2 CE1	4 CE5								3 CE4		
3.1.1.73	feruloyl_esterase	1 GH10											
3.2.1.8	endo-1,4-β-xylanase	6 GH11	10 GH10								1 GH10		
3.2.1.37	xylan_1,4-β-xylosidase	1 GH5	1 GH3	2 GH43									
3.2.1.55	α-N-arabinofuranosidase	1 GH43											
3.2.1.131	xylan_α-1,2-glucuronosidase	1 GH115											
3.2.1.151	xyloglucan-specific_endo-β-1,4-glucanase	3 GH74											
Pectin													
3.1.1.11	pectinesterase	1 CE8									2 CE8		
3.2.1.15	polygalacturonase	2 GH28					1 GH28				6 GH28		
3.2.1.22	α-galactosidase						2 GH27		1 GH36				
3.2.1.23	β-galactosidase	1 GH35					4 GH35		1 GH35		1 GH2		
3.2.1.40	α-L-rhamnosidase												
3.2.1.67	galacturan_1,4-α-galacturonidase	1 GH28									4 GH28		
3.2.1.89	arabinogalactan_endo-β-1,4-galactanase	2 GH53									1 GH53		
4.2.2.2	pectate_lyase	1 PL1	1 PL10	4 PL3									
4.2.2.10	pectin lyase										4 PL1		
4.2.2.-	rhamnogalacturonan lyase	2 PL4											
Starch													
3.2.1.1	α-amylase	1 GH13				2 GH1							
33.2.1.3	glucan_1,4-α-glucosidase	1 GH15				1 GH15	4 GH15		1 GH15	1 GH15	GH15		
3.2.1.20	α-glucosidase	1 GH31	1 GH13			3 GH31	1 GH31		1 GH31	1 GH31	1 GH31		
3.2.1.28	α,α-trehalase	2 GH37				3 GH37	1 GH37		1 GH37	1 GH37	2 GH37		
Mannan													
3.2.1.24	α-mannosidase	2 GH38				1 GH38	3 GH38		1 GH38	1 GH38			
3.2.1.25	β-mannosidase	2 GH2				1 GH2	1 GH2			1 GH2			
3.2.1.78	mannan_endo-1,4-β-mannosidase	4 GH5	2 GH26			1 GH5	1 GH5						
3.2.1.113	mannosyl-oligosaccharide_1,2-α-mannosidase	3 GH47				4 GH47	8 GH47	6 GH47	4 GH47	1 GH92	5 GH47		
Chitin													
3.2.1.14	chitinase	7 GH18				2 GH182	2 GH18	2 GH18	3 GH18		12 GH18	1 GH19	
3.5.1.41	chitin_deacetylase	5 CE4				6 CE4	1 CE4	8 CE4	10 CE				
43.2.1.52	β-N-acetylhexosaminidase	1 GH20				1 GH20	11 GH20	1 GH20					
3.2.1.132	chitosanase					1 GH8	1 GH46						
3.2.1.165	exo-1,4-β-D-glucosaminidase						1 GH9

**Table 3:** Comparative analysis of enzymes from four phyla of early-diverging, zoosporic fungi. Species from *Neocallimastigomycota*, *Blastocladiomycota*, and *Cryptomycota*. The left hand column lists the enzyme functions found in the respective genomes, predicted to EC enzyme classification as well as EC functional description by peptide-based functional analysis (herein the peptide-based program, Hotpep). The table is modified from, and based on the data published in, Lange et al. (2018). The EC functions are grouped according to the type of substrate the predicted enzymes act on: plant cell wall carbohydrates (cellulose, hemicellulose, and pectin) and storage and fungal cell wall carbohydrates (starch, mannan, chitin)

		Neocallimastigomycota										Blastocladiomycota		Cryptomycota
EC #	Function description	*Piromyces finnis*			*Anaeromyces robustus*			*Neocallimastix californiae*				*Allomyces macrogynus*	*Catenaria anguillulae*	*Rozella allomycis*
Cellulose														
3.2.1.4	endo-1,4-β-D-glucanase	11 GH9	12 GH45	22 GH5	9 GH9	11 GH45	20 GH5	12 GH9	20 GH45	1 GH26	40 GH5			
3.2.1.21	β-glucosidase	14 GH3	10 GH1		14 GH3	6 GH1		42 GH3	16 GH1					
3.2.1.91	cellulose1,4-β-cellobiosidase (non-red_end)	20 GH6			11 GH6			25 GH6						
3.2.1.176	cellulose_1,4-β-cellobiosidase_(red_end)	12 GH48			6 GH48			20 GH48						
1.1.99.18	cellobiose_dehydrogenase_(acceptor)													
1.1.99.29	pyranose_dehydrogenase_(acceptor)													
Hemi-cellulose														
3.1.1.6	acetylesterase							2 CE16				1 CE16	1 CE16	
3.1.1.72	Acetylxylan_esterase	1 CE2	2 CE4	9 CE6	11 CE2	12 CE4	11 CE6	5 CE2	22 CE4	14 CE6				
3.1.1.73	feruloyl_esterase	4 CE1			11 CE1			6 GH10						
3.2.1.8	endo-1,4-β-xylanase	36 GH11	18 GH1	1 GH8	29 GH11	14 GH1	2 GH8	22 GH11	1 GH3	1 GH8	46 GH10	4 GH11		2 CE4
3.2.1.37	xylan_1,4-β-xylosidase	2 GH43			1 GH120	6 GH43		2 GH3	9 GH43					
3.2.1.55	α-N-arabinofuranosidase	8 GH43			4 GH43			13 GH43						
3.2.1.131	xylan_α-1,2-glucuronosidase	2 GH115			1 GH115			7 GH115						
3.2.1.151	xyloglucan-specific_endo-β-1,4-glucanase	1 GH74			1 GH74			4 GH74						
Pectin														
3.1.1.11	pectinesterase													
3.2.1.15	polygalacturonase							1 GH28				3 GH28		
3.2.1.22	α-galactosidase													
3.2.1.23	β-galactosidase	1 GH2			1 GH2			7 GH2						
3.2.1.40	α-L-rhamnosidase				1 GH78			1 GH78						
3.2.1.67	galacturan_1,4-α-galacturonidase													
3.2.1.89	arabinogalactan_endo-β-1,4-galactanase	1 GH53			1 GH53			3 GH53						
4.2.2.2	pectate_lyase	4 PL1	3 PL3		3 PL1	1 PL3		19 PL1	6 PL3				1 PL3	
4.2.2.10	pectin lyase													
2 PL14.2.2.-	rhamnogalacturonan lyase													
Starch														
3.2.1.1	α-amylase				1 GH13								3 GH13	
3.2.1.3	glucan_1,4-α-glucosidase											8 GH15	2 GH15	
3.2.1.20	α-glucosidase	2 GH31	1 GH13		5 GH31			9 GH31	1 GH13			3 GH31	1 GH31	2 GH31
3.2.1.28	α,α-trehalase	1 GH37			1 GH37			1 GH37				2 GH37	1 GH37	2 GH37
Mannan														
3.2.1.24	α-mannosidase	1 GH38			2 GH38			1 GH38				5 GH38		1 GH38
3.2.1.25	β-mannosidase													
3.2.1.78	mannan_endo-1,4-β-mannosidase	1 GH5	3 GH26		4 GH5	2 GH26		7 GH5	7 GH26			2 GH26		
3.2.1.113	mannosyl-oligosaccharide_1,2-α-mannosidase	1 GH47			4 GH47			6 GH47				2 GH47	2 GH47	3 GH47
Chitin														
AA11	LPMO													2 AA11
3.2.1.14	chitinase	6 GH18						16 GH18				6 GH18	6 GH18	2 GH18
3.5.1.41	chitin_deacetylase	6 CE4			3 CE4			18 CE4				5 CE4	4 CE4	
3.2.1.52	β-N-acetylhexosaminidase											2 GH20	1 GH20	
3.2.1.132	chitosanase							1 GH8						
3.2.1.165	exo-1,4-β-D-glucosaminidase													

## Enzyme families and function in zoosporic fungi

### Cellulose- and hemicellulose-degrading enzymes in early-diverging fungi

A comparative overview of cellulases and hemicellulases plus relevant LPMO (lytic polysaccharide monooxygenase, auxiliary activity enzymes) across all four fungal phyla of early-diverging zoosporic fungi is presented in Tables 2 and 3. Based on the genome-sequenced species studied in Lange et al. (2018), it appears that there are two prominent groups of biomass-degrading fungi: the anaerobic rumen fungi and the terrestrial soil-inhabiting, aerobic fungi *R. rosea* and *Rhizoclosmatium globosum* (Olive 1983; Mondo et al. 2017). Furthermore, both *Caulochytrium protosteloides* (*Caulochytriales*, *Chytridiomycota*) (Powel 1981; Ahrendt et al. 2018) and *Gonapodya prolifera* (*Monoblepharidales*, *Chytridiomycota*) have a rich CAZyme profile. It is characteristic for the lignocellulolytic species analyzed here that the beta-1,4-endoglucanase activity (EC 3.2.1.4) is represented by multiple protein families: GH5, GH9, and GH45 in anaerobic rumen fungi; GH9 and GH45 in *G. prolifera*; and GH5, GH7, GH9, and GH45 in *R. rosea*. *Gonapodya prolifera* has a strong portfolio of enzymes targeting cellulose and hemicellulose, and this species also has the most diverse spectrum of pectin-active enzymes. The weakest profile of cell wall–degrading enzymes is found within the *Blastocladiomycota* and the *Cryptomycota* (Tables 2 and 3). The recently published genomes of zoosporic fungi *R. globosum* and *Caulochytrium protosteloides* (Table 1) are shown to have an even higher number of CAZymes than *R. rosea*. Furthermore, the exceptionally high numbers of CAZymes for the rumen fungi support the premise that the rumen fungi are strong biomass degraders.

Chang, Berbee, and coworkers have described *G. prolifera* to be rich in the diversity of pectin-active enzymes (Chang et al. 2015), having 21 pectinase genes covering the major types of pectinase activities. Lange et al. (2018) using peptide-based annotation (Busk and Lange 2012; Busk et al. 2017), in addition to confirming the findings of Chang et al. (2016), identified *R. rosea* as a rich source for pectin-degrading enzymes. The rumen fungi, especially *N. californiae*, likewise, have been shown to have a rich portfolio of pectinases (Solomon et al. 2016; Haitjema et al. 2017). Sprockett and Piontkivska (2011) studied the evolution and versatile roles of the GH28 enzymes involved in breaking down the pectin backbone, and Benoit et al. (2012) gave a broader description of fungal enzymes involved in pectin degradation to include enzymes that hydrolyze pectin sidechains. There is a plethora of studies on fungal cellulases. Seen in light of evolution of the fungal secretome and the heterogeneous composition of pectin, pectin-degrading enzymes deserve more studies.

The classical description of *R. globosum* is that it lives on insect exuviates. Yet, the CAZymes functionally annotated from its genome include pectin-degrading enzymes (Table 2), suggesting that it is able to convert other carbohydrate substrates besides chitinous materials. The description of substrate affinity of this species as being primarily chitin could stem from the observation that this species is easily isolated from soil samples baited with insect chitin skeletons. But according to its CAZyme content, this species is not confined to metabolizing chitin.

The distribution of cellulases and hemicellulases of the six aerobic chytrids is shown in Table 2. Of the 13 different families of hemicellulose-active enzymes (representing eight different biochemical activities) found in *R. rosea*, as many as eight are not found in other species of *Chytridiomycota*, *Blastocladiomycota*, and *Cryptomycota* examined, see Tables 1 and 2. A GH6 cellobiohydrolase (EC 3.2.1.91) for *R. rosea* shown here is unique among *Chytridiomycota.* Such data can be used to support two different hypotheses: all other aerobic chytrids reviewed here have lost this set of hemicellulases (and cellulases) or that *R. rosea* has acquired this spectrum of plant cell wall–degrading enzymes through horizontal gene transfer during its adaptation to terrestrial life in degrading plant biomass in agricultural soils. Supporting the gene loss hypothesis over horizontal gene transfer is that *R. rosea* shares some of its unique enzymes with the anaerobic rumen fungi. However, Haitjema et al. (2017) concluded that several catalytic CAZyme domains of rumen fungi had originated via horizontal gene transfer from gut bacteria. The evolutionary importance of horizontal gene transfer in zoosporic fungi have been further supported by the recent studies of Duarte and Huynen (2019) and Murphy et al. (2018).

The 12 species of zoosporic fungi studied by Lange et al. (2018) show similar patterns of enzymes involved in the degradation of the storage and fungal cell wall materials (starch, mannan, and chitin). As shown in Tables 2 and 3, a set of three enzymes is found to be present in all species: GH31 alpha-glucosidase (EC 3.2.1.20) and GH37 trehalase (EC3.2.1.28) for starch, and GH47. Furthermore, the cell wall chitin–modifying enzyme GH18 chitinase (EC 3.2.1.14) is also found in all species studied across the four phyla of zoosporic fungi. With regard to the types of enzymes that target storage materials and fungal cell wall, it is also striking that the *Blastocladiomycota* and *R. allomycis* (*Cryptomycota*) have a rather rich, typical fungal profile of enzymes degrading starch, mannan, and chitin (Table 2). Interestingly, a key enzyme of fungi in general, the cell wall–modifying beta-glucanase, is in these studies not found in the *Blastocladiomycota*, *B.dendrobatidis*, and *R. allomycis.* Lange et al. (2018) reported beta-glucanase to be present broadly among the other early-diverging, zoosporic phyla. The enzyme groups in early-diverging fungi about which we still have only limited molecular information are the proteases and the lipases. Most of the information about proteases have been derived from studies of the pathogenic species, especially *B. dendrobatidis* (Berger et al. 2016, Symonds et al. 2008). The proteases recorded have been described as being trypsin, chymotrypsin-like, or keratinolytic. (For the total number of proteases found, see the right-hand column in Table 1.)

## Cellulosomes and secretome composition

The studies by Gruninger et al. (2014, 2018) and Solomon et al. (2016) provided not just overviews but also new knowledge on the enzymes of rumen fungi, describing the large, comprehensive array of biomass-degrading enzymes in zoosporic fungi. Multiple studies hinted that biomass-degrading enzymes in early-diverging fungi are organized in large complexes (Wilson and Wood 1992; Dijkerman et al. 1996, 1997). Harhangi et al. (2003) studied a GH6 cellobiohydrolase enzyme of *Piromyces*, showing the importance of the “high molecular mass complex called cellulosomes.”

A significant step towards understanding the evolution, structure, and function of the cellulosomes of rumen fungi was provided by Haitjema et al. (2017). The basis for the focused studies of the cellulosome structure study of rumen fungi was the high-quality genome sequences from three rumen fungal species representing three different genera: *N. californiae*, *Piromyces finis*, and *Anaeromyces robustus* (Solomon et al. 2016; Haitjema et al. (l.c.). In attempts to identify components of cellulosome across the three rumen fungi studied, they found as many as 312 non-catalytic dockerin domains, numerous CAZymes, and 95 scaffoldins. The rich array of CAZymes found to be part of the fungal cellulosome was also shown in their study to include GH3 and GH45. These two enzymes had not previously been reported to be integral parts of either the fungal or the bacterial cellulosome. That GH3 protein as an integral part of the cellulosome (Haitjema et al. 2017) is significant in that it enables the rumen fungi to break down the plant polymers to monomeric sugars extracellularly for localized import. Still, the observation in the laboratory is that rumen fungi grow poorly on monomeric sugars in culture. They grow much better on cellobiose than on glucose.

Through sequence similarity studies, Haitjema and co-workers were able to arrive at the following conclusion: the scaffoldin domains of the cellulosome has no similarity to the dockerin and scaffoldin domains of bacterial cellulosome, whereas the CAZy enzymes of the fungal cellulosome showed high similarity to bacterial enzymes. The fungal cellulosome appears to be a chimeric structure that integrates the fungal-derived structural proteins with several bacterial-derived catalytic domains, while also integrating carbohydrate-binding modules (CBMs) and a membrane-spanning anchor to the cell membrane of the organism (Haitjema et al. 2017). Furthermore, Haitjema et al. (l.c.) concluded that the cellulosome has evolved independently twice—once by fungi and once by bacteria—in the rumen habitat. An evolutionary development of a structure of such complexity, highly optimized, structurally, and functionally organizing multiple enzymes into a cellulosome, must (in order to have been selected for in evolution) have offered significant advantages in fitness. As the rumen is an aquatic environment, cellulosomes could offer advantage in reducing diffusion of hydrolyzed oligosaccharides and monosaccharides as well as in reducing diffusion of enzymes. The latter effect is possibly achieved through the transmembrane helix, interpreted to anchor the cellulosome to the host cell (see Haitjema et al. 2017, Fig. 1).

The early study of Fanutti et al. (1995) showed that the CAZyme dockerin domain can bind to the cellulosome of several species of rumen fungi. This observation was confirmed and expanded by Haitjema et al. (2017) by providing evidence that purified single dockerin domains and scaffoldin fragments from three species (belonging to three different genera) of rumen fungi could bind in all combinations across the three species. Similar studies of bacterial cellulosomes showed that dockerin and scaffoldin binding (in the bacterial community) is species-specific. Haijtema and coworkers (l.c.) concluded that this promiscuity of fungal dockerins and fungal scaffoldins give the fungi one more selective advantage over bacteria in the rumen environment. It is therefore of crucial importance to include the rumen fungi (and other eukaryotes) in rumen microbiome studies in order to understand the function of the rumen.

Many CAZymes of rumen fungi are apparently not integrated in cellulosomes as they are found not to contain CBM10, dockerin. About 70% of CAZymes of rumen fungi have CBM domains, and half of them are associated with CBM10. In other words, ~ 35% of the CAZymes of anaerobic fungi have dockerin domains and > 60% without (Gruninger et al. 2018). It would be highly interesting to uncover whether expansins and swollenins play a role in overcoming the recalcitrance of crystalline cellulose in the feed biomass, where there are no LPMOs in rumen fungi. Regarding the anchoring of the cellulosome to the organismal cell membrane, this in itself could provide a significant advantage, as the anchoring may have the effect that the cellulosomes become less prone to being washed out with the rumen fluid. The rumen fungi are efficiently anchored by their rhizoids to the biomass particles (named rumen solids), and the cellulosome is anchored to the cell membrane.

## Recombinant production of enzymes of zoosporic fungal origin

Separated early in evolution, enzymes from early-diverging fungi could be expected to be difficult to produce recombinantly in hosts typically used for production of Dikarya (ascomycetous and basidiomycetous) enzymes. However, in our experience as well as that reported in published investigations, recombinant production does not appear to present a real obstacle. Carbohydrate-active enzymes from rumen fungi have been produced successfully (Harhangi et al. 2003). Enzymes from aerobic chytrids have been produced in the yeast *Saccharomyces cereviciae* (cDNA expression libraries with *S. cereviciae* as screening host to detect secreted and active cellulases). Beta-1,4-endoglucanase has been successfully produced in *Pichia pastoris* (Pilgaard 2014; Lange et al. 2018). Furthermore, Huang et al. (2018) recombinantly produced both GH11 and GH43 proteins from *R. rosea* in *P. pastoris*. Production of enzymes from rumen fungi has also been shown to be possible for genes from *Piromyces* and *Orpinomyces* (Gruninger et al. 2014). However, the feasibility of recombinant production of enzymes from early-diverging fungi in filamentous fungi (with high yields in *Trichoderma* spp. or *Aspergillus* spp*.*, opening for commercial scale production) needs more studies.

## Comparing the enzyme secretome composition of different types of lifestyles

### Specialization to the anaerobic eukaryote lifestyle

The rumen fungi are exceptional in the fungal kingdom not only because they are zoosporic and belong to the early-diverging lineages but also because they are anaerobic. Anaerobic eukaryotes (here fungi) have not been described from many habitats, and anaerobic habitats in general have not been intensely studied. Occurrence of anaerobic eukaryotes in more gut systems from a wide range of animals is among the discoveries that we should look out for in microbiome studies. Gruninger et al. (2014) reviewed the characteristics of the anaerobic rumen fungi. The major adaptation to a life without oxygen is the presence of the specialized organelles, hydrogenosomes, which couple the metabolism of glucose to cellular energy production without the need for oxygen. Among the other features related to the anaerobic eukaryote lifestyle is posttranslational fucosylation. With regard to enzyme secretome composition, the most striking difference to the aerobic chytrids is that the rumen fungi do not have any LPMOs. Being obligate anaerobic, rumen fungi are not expected to have oxygenases. Yet, the LPMO enzymes have been shown to function without oxygen (Hegnar et al. 2018). The second interesting difference in enzyme composition is that the anaerobic rumen fungi do not have GH7, the reducing-end cellobiohydrolase. However, they possess GH48 proteins that have the same function as GH7 cellobiohydrolases of aerobic zoosporic fungi.

### Pathogenic vs saprophytic

Joneson et al. (2011) studied the genomic transition to pathogenicity in chytrid fungi by comparing *H. polyrhiza* and *B. dendrobatidis*. They found these two species to be closely related phylogenetically but to be significantly different when it came to secretome composition. Significant changes in the CAZyme portfolio have taken place in the specialization/transition to a pathogenic lifestyle. *Batrachochytrium dendrobatidis* was observed to be enriched for proteases, lipases, and effector genes (Joneson et al. 2011). These authors concluded that the protease gene family expansion predated the emergence of the *B. dendrobatidis* epidemic as a threatening global disease of amphibians.

The specialized endoparasite *R. allomycis* (*Cryptomycota*) has been shown to have a relatively large proteome (> 6350 proteins) for an endoparasite, larger than the proteome of baker’s yeast *S. cereviciae*. The suggested explanation for this is that *R. allomycis* has developed multiple proteins for protein-protein interaction, for manipulation of its fungal host, and possibly also for recycling of host proteins. These developments have enabled the fungus to live by mobilizing nutrients from its hosts, and therefore, it does not need the CAZyme complement required for degradation of cellulose and hemicellulose (Table 1; James et al. 2013).

## Evolution of the zoosporic fungal enzyme secretome

Comparative and phylogenetic studies of the secretome composition and evolution of zoosporic fungi (most of which are aquatic) could provide a new dimension to basic studies of evolution of the ocean (Dunn 2013, in Current Biology) and provide additional perspectives to the attempts at a consistent phylogenetic characterization of fungi provided by Ebersberger et al. (2012).

Chang et al. (2015) identified pectin-degrading enzymes to be a more adequate measure for estimating fungal evolution in geological time based on the following rationale. Cellulose is found among many different types of organisms, which indicates that metabolizing capacity to degrade cellulose cannot be used to date evolutionary events. However, the initial occurrence in geological time of pectin-containing plants has been estimated at 750 million years ago, meaning that fungi specialized in degrading pectin-containing organic substrates cannot date further back than 750 million years. This intriguing rationale was unfolded and illustrated by studying *G. prolifera.* This approach, however, could also be applied to the interpretation of the secretome composition evolution described for the pectinase-rich *R. rosea*. Using this as a baseline, the transition of aerobic chytrids (here exemplified by *R. rosea*) to terrestrial life therefore cannot date back further than 750 million years.

James et al. (2013) observed that *Cryptomycota* and *Microsporidia* are united in sharing signatures of parasitism and in having chitinous cell walls. The comparative enzyme secretome studies of Lange et al. (2018) showed that *R. allomycis* shares a core set of enzymes with species of all other phyla of early-diverging fungi. Furthermore, Lange et al. (2018) (and Lange et al., in publication) showed that the AA11 LPMO was also found in *Cryptomycota* as well as in other fungal phyla, though not in Microsporidia, while the protozoan *Dictyostelium discoideum* was also shown to have an AA10 LPMO gene, implying that AA10 dates back prior to separation of Amoebozoae. An additional perspective to this finding is that peptide-based functional annotation across the entire fungal kingdom suggests that AA10 could have been acquired by horizontal gene transfer at different points during fungal evolution and not by common descent (Lange et al., published at 2nd LPMO meeting, Marseilles, France, 2018). The evolutionary importance of horizontal gene transfer in zoosporic fungi have been further supported by the recent studies of Duarte and Huynen (2019) and Murphy et al. (2018).

**Fig. 2:**
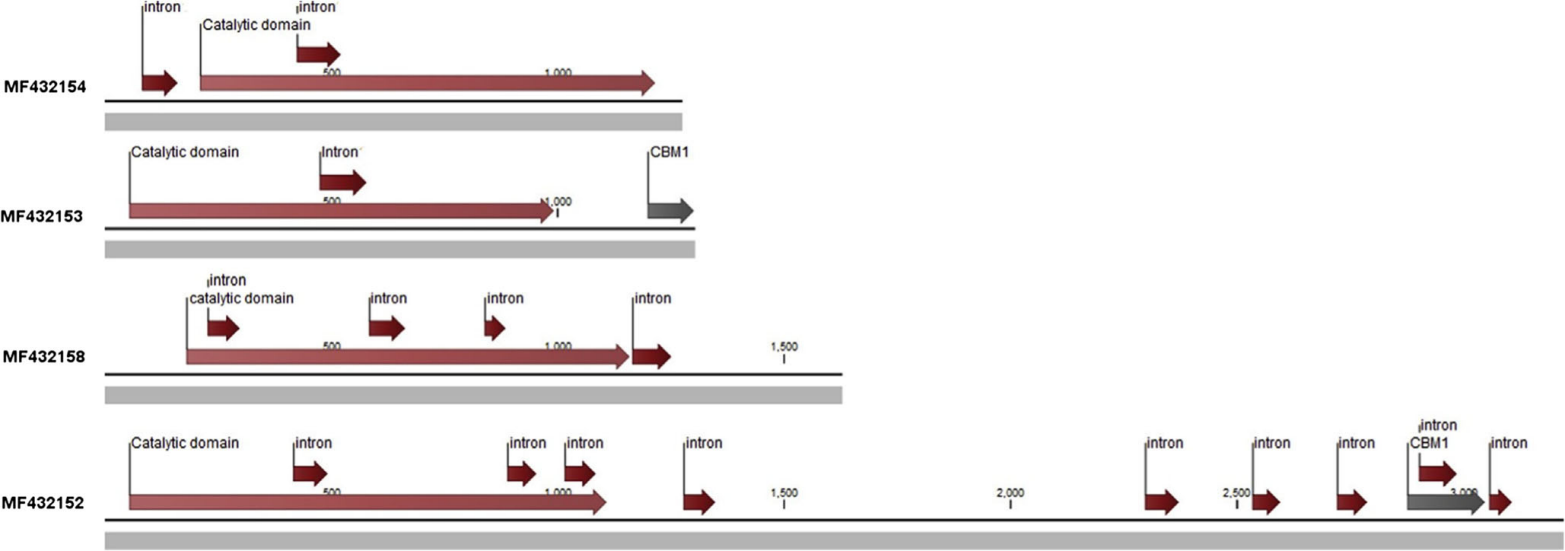
Comparing gene structure of different copies of GH5 (EC 3.2.1.4), found in the *R. rosea* genome. The uppermost GH5 variant of *R. rosea* has one intron in the catalytic domain and no CBM. The other GH5 gene variants have a more typical fungal gene structure: a CBM1 domain and up to many (here 9) introns and additional protein domain inserts. The gene structure of the bacterial-related *R. rosea* GH5 has a gene structure more closely related to bacterial genes. From Lange et al. (2018), with courtesy of Fungal Biology Reviews

Lange et al. (2018) observed that biomass-degrading enzymes (especially endo-glucanases and endo-xylanases) are found in several variants (not just similar copies) in the genomes of biomass-degrading zoosporic fungi. Comparative analysis of gene structure of such “copies” of GH5 beta-1,4-endoglucanase (EC 3.2.1.4) from *R. rosea* revealed significant differences (Fig. 2). The *R. rosea* GH5 endoglucanase gene copy, which in the phylogenetic tree was found embedded in the bacterial clade, has a bacterial-like gene structure. One interpretation could be that genes of metabolic enzymes of both fungal origin and bacterial origin (such as endo-glucanases), under strong evolutionary pressure, can be acquired and maintained in the same genome. Recent studies by Duarte and Huynen (2019) and Murphy et al. (2018) give evidence that horizontal gene transfer is an important factor in evolution of Neocallimastigomycetous fungi. However, more comparative studies of gene structure will have to be conducted before conclusions can be drawn for the zoosporic fungi as such.

**Fig. 3:**
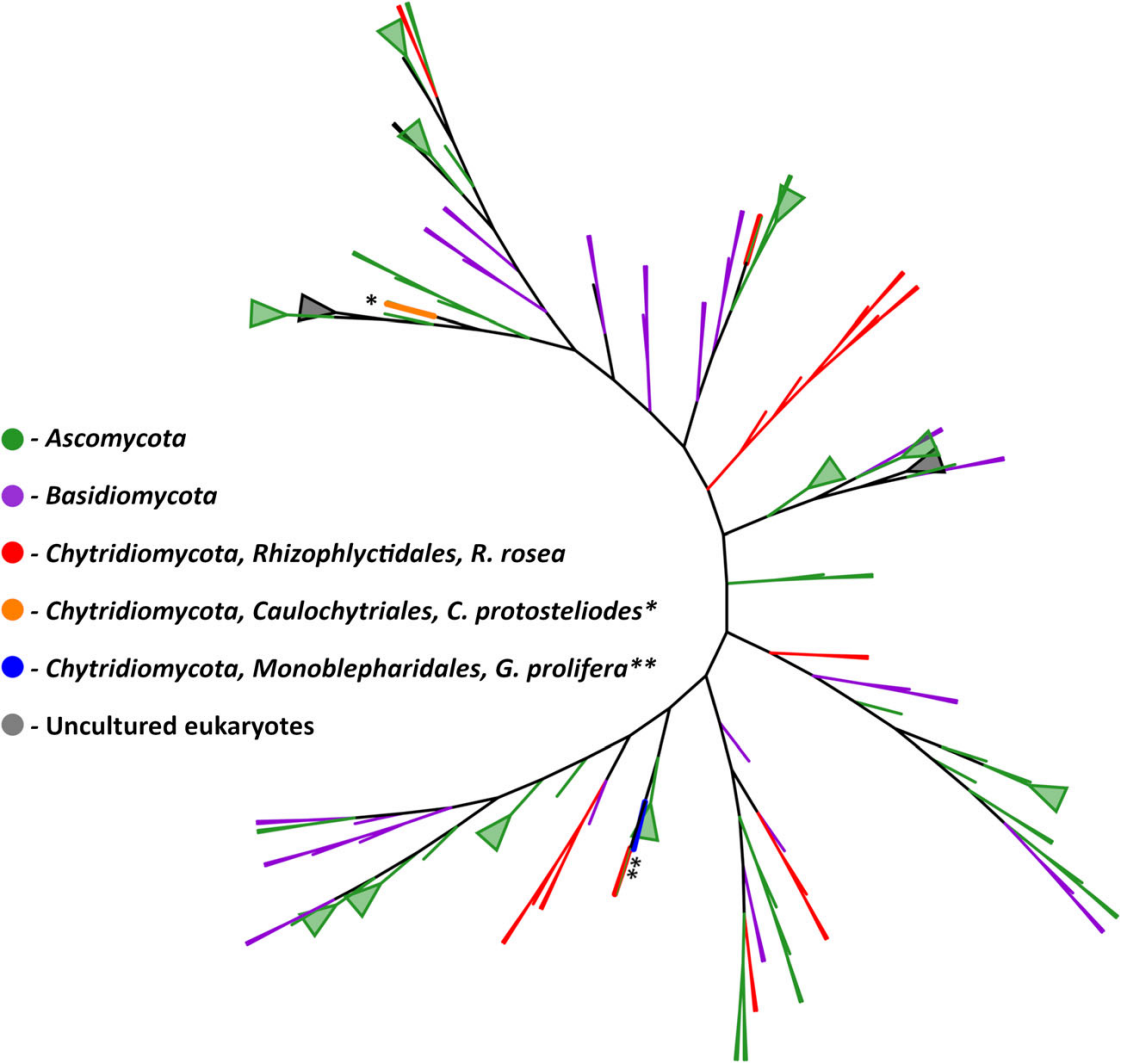
Phylogenetic tree of fungal AA9, placing diversity of zoosporic AA9 in a fungal kingdom perspective. A maximum likelihood phylogenetic tree of CAZy-listed AA9 sequences. Triangles represent collapsed clades of similar LPMO AA9 sequences from the same phylum. To generate the tree, amino acid sequences were downloaded from GenBank (www.ncbi.nlm.nih.gov/), trimmed to the predicted catalytic domains using dbCAN (http://bcb.unl.edu/dbCAN2/), and aligned in MAFFT (www.ebi.ac.uk/Tools/msa/mafft/). This alignment was used to generate the final tree in RaxML-blackbox, available on the CIPRESS server (https://www.phylo.org/). Branch colors: green = *Ascomycota*; purple = *Basidiomycota*; red = *Rhizophlyctis rosea*, *Rhizophlyctidiales*, *Chytridiomycota*; orange = *Caulochytrium protosteloides*, *Caulochytriales*, *Chytridiomycota* (marked by single asterisk); blue = *Gonapodya prolifera*, *Monoblepharidales, Chytridiomycota* (marked by double asterisks). The tree is an updated version of the AA9 phylogenetic tree of Lange et al. (2018), with courtesy of Fungal Biology Reviews

Another analysis of diversity among several “copies/variants” of same gene (here the AA9 LPMO) is shown in Fig. 2. As appears from Fig. 3, the several AA9 genes in *R. rosea* (> 20 in all) are placed in clades that include ascomycetous and basidiomycetous AA9. However, the *R. rosea* genome also includes a unique clade of six AA9 genes that are shown to belong to a separate clade from the AA9 of the *Dikarya* fungi. It is an example that unique enzymes, of both evolutionary and biomass-conversion relevance, are found among early-diverging fungi.

## Application potential

Based on the results regarding successful heterologous production of enzymes from zoosporic fungi, it is tempting to suggest that it would be feasible to produce, in existing fungal production hosts, a blend of enzymes derived from early-diverging fungi. Furthermore, an all bacterial-derived biomass-degrading blend could be made possible by producing all needed enzymes in a bacterial host by replacing GH7 with the rumen fungi-derived (or bacterial) GH48. Similarly, it would be highly interesting to explore how an optimized blend could be produced that relies exclusively on exploiting the cellulosome structure, adding the swollenin and expansin, or on the flip side to clarify whether these enzymes only work efficiently under anaerobic conditions, suitable for anaerobic digesters but not much else.

As reviewed above, Chang et al. (2015) showed that at least some of these enzymes (pectinases) are relatively recent innovations. The argument maybe that they reside in environment very different from the ascomycetes and basidiomycetes, where most of the studies on CAZymes have focused on, and that they have likely evolved different properties. As for efficiency of their enzymes, that is debatable. For applied purposes, we have worked primarily with terrestrial ascomycetes and basidiomycetes because they are found in nature to be the major decomposers of terrestrial biomass.

Due to their early divergence, the enzymes and the secretome composition of zoosporic fungi have had longer evolutionary time to develop independently from the other groups of fungi and dynamically optimize their efficiency in metabolizing substrates in their niche environments. The early-diverging fungi (and their enzymes) have come a long way, and the fact that they are here today is a sign of competitiveness and ability to adapt to new conditions. The question is “could this optimized portfolio of biomass-degrading enzymes be used to generate value in the rapidly growing business of making higher value products?”

The application potential of enzymes from zoosporic fungi, in all their diversity and novelty, seems promising from the studies reviewed here. So far, no commercialization has taken place though there have been many interesting R&D attempts that have provided important information and lessons. Morrison et al. (2016) published a suggestion for a biomass-degrading, defined blend of four components from the anaerobic fungus, *Orpinomyces* sp., strain C1A. The suggested blend consisted of a beta-1,4-endoglucanase (EG5/GH5), a GH6 (Cel6A) cellobiohydrolase, an endoxylanase (GH11), and a multifunctional beta gluco-, xylo-, and galactosidase. Applying this enzyme cocktail to pretreated corn stover or switchgrass resulted in 65–77% saccharification, depending on the substrate recalcitrance. Morison and co-workers further showed that adding fungal swollenin improved the hydrolysis by up to 7%, apparently through boosting both glucan and xylan hydrolysis. Onifade and co-workers, as early as in 1998 (Onifade et al. 1998), made clear guidance for how rumen fungi could be used for nutritional improvement of feather and other keratinaceous waste for use in animal feed. Prochazka et al. (2012) published an attempt to use rumen fungi to boost biogas production

Regarding applied use of chytrid secretome, to the best of our knowledge, no attempts have been made to use either individual enzymes or a blend of enzymes derived from the aerobic chytrid *R. rosea*. It could be worth trying a blend of enzymes, which mimics the potent secretome blend of, e.g., *R. rosea* secretome (or secretome of other specialized, biomass-degrading zoosporic fungi), to be used as a commercial blend, produced in, e.g., *Trichoderma* spp*.* Such a blend would be very interesting to study, albeit highly complex. *Rhizophlyctis rosea* has a portfolio of 20 AA9 LPMOs expressed at the same time. Another approach of relevance for applied use of chytrid biomass–degrading LPMOs is to use the diversity (20 LPMOs possibly representing different roles/functions) and thus valorize the information provided by CUPP analysis (Barrett and Lange 2019) that assigns the enzymes of *R. rosea* to different CUPP-defined groups, most probably reflecting different functions or function-related features. Moreover, a community of microorganisms is usually involved in the efficient decomposition of biomass. The community secretomes maybe “optimized” to breakdown efficiently selected components of complex biomass (de Vries et al. 2017; Mäkelä et al. 2018).

Climate change mitigation has brought cattle production into the spotlight, due to its high emission of methane. With this as a driver, knowledge gained from the rumen fungi secretome could be used as basis for possibly developing new feeding and breeding strategies for reduction of ruminant methane emission. Such strategies could exploit the potential of prebiotic and probiotic effects of specific types of feeding materials that might possibly modify rumen microbiome compositions towards lower methane emission by inhibiting or outcompeting the methanogenic archaea.

## Future perspectives

Relevant evolutionary information can be obtained by comparative analysis of genomes, across the four zoosporic, early-diverging fungal phyla, by taking advantage of valuable functional annotation platforms, such as the well curated CAZyme database (CAZy.org), the HMM models (Mistry et al. 2013), CUPP (Barrett and Lange 2019), dbCAN2 (Zhang et al. 2018), Diamond (Buchfink et al. 2015), and SACCCHARIS (Jones et al. 2018). Furthermore, biological insight on the secretome can be reinforced by conducting activity screening and mass-spectrometry-based proteomics analysis of culture broths of key species. Results from these experimental investigations would reveal the identity of enzymes in the secretome and the approaches adopted by different fungi in interacting with their environment, host, and/or substrates. Such information can contribute to the knowledge on the evolution of the early-diverging, zoosporic fungi and provide additional clarity to the basal evolutionary roots of the fungi.

High priority should also be given to expanding the studies of secretome composition beyond the CAZymes to include in detail the proteases and the lipases. The focus on enzyme discovery should be expanded to include next-level information and understanding of the synergies among the various types of enzymes present in the fungus/substrate and fungus/host secretomes. Likewise, there should be greater emphasis on the possible mechanisms of interaction and synergies encountered in the secretome of gut microbiomes during interaction of fungi with both host and substrates.

Gut microbiome metagenomics of a broader spectrum of insects and nematodes should be explored to investigate whether *Neocallimastigomycetes* (and maybe other zoosporic fungi) are also found in other digestive systems beyond the herbivores. Further studies are also needed on the interaction of zoosporic fungi with other types of organisms; for example, the observation of negative impacts on rumen fungi of protozoae and bacteria (see Gruninger et al. 2014).

Enzyme secretome composition and enzyme interactions with the host could be used to expand our understanding of what caused the amphibian epidemic disease. Such studies can provide a basis for understanding the threatening fungal epidemic diseases of snakes (*Onygenales*) and bats (white nose, caused by *Geomyces destructans*). Improved understanding of fungal epidemics in wild animals can help prepare us for possible higher occurrences of fungal diseases in humans. Interestingly, well-documented examples of shifting trends in epidemics have been documented in human pathogen dynamics (Smiths and Guegan 2010; Woolhouse and Gaunt 2007; Hoskisson and Trevors 2010). Animal studies, including bioimaging-enabled resolution in time and space of enzyme activities in pathogen/host and parasite/host interactions, could be of special interest in this endeavor.

A full understanding of the roles of the different types of organisms present in the rumen micobiome has not yet been achieved. In future studies, it is important to ensure that the sampling, extraction, sequencing, and annotation do not introduce bias to the representation of different groups of microorganisms, prokaryotes, and eukaryotes. Among the concerns are the sampling of rumen microbiome for metagenome sequencing for functional studies. Populations of fungi and bacteria fluctuate rapidly post feeding. Thus, special care needs to be taken to ensure that neither prokaryotes nor eukaryotes is positively or negatively biased in the applied experimental procedures and the bioinformatics analysis.

It is of urgent importance that we improve our overall understanding of the function of the rumen microbiome. Otherwise, attempts to identify possible breeding or feeding strategies for a more climate friendly cow are inherently going to fail because we do not grasp the full picture of rumen function. The question is as follows: could rumen microbiome function be modified by stimulating the rumen fungi through a combined feeding and breeding effort guided and monitored by microbiome studies? Could such targeted changes in the rumen microbiome lead to reduction of methane emission? Further, to be even more ambitious, we could take the evolutionary studies of the secretome composition and activities of zoosporic fungi to a new level. This may be achieved by comparing their secretome composition (and evolution) to the secretome of other groups of organisms, such as *Microsporidia*, *Mucoromycota*, and *Zoopagomycota*. Similarly, it would be interesting to compare the fungal secretome with the secretome of non-fungal, zoosporic groups of organisms that are so similar to fungi in their ecology, habitat occurrence, substrate interaction, and physiology (Gleason et al. 2018). In such comparisons, additional understanding of horizontal gene transfer in the evolution of the (apparently highly dynamic) secretome of aquatic zoosporic organisms could be achieved by pursuing deeper level studies of such events. The evolutionary importance of horizontal gene transfer in zoosporic fungi have been further supported by the recent studies of Duarte and Huynen (2019) and Murphy et al. (2018).
